# Reconstruction With Ipsilateral Fibula Transfer With Pasteurized Bone After Excision of Bone Sarcoma of the Tibia

**DOI:** 10.1080/13577140400017794

**Published:** 2004

**Authors:** Toshifumi Ozaki, Kazuo Fujiwara, Toshiyuki Kunisada, Tatsuo Ito, Akira Kawai, Hajime Inoue

**Affiliations:** 1 Department of Orthopaedic Surgery Okayama University Hospital 2-5-1 Shikata-cho Okayama 700-8558 Japan; 2 Department of Orthopaedic Surgery National Cancer Center Hospital Tsukiji, Chuo-ku Tokyo 104-0045 Japan

## Abstract

We report a technique of implantation of the ipsilateral vascularized fibula with pasteurized recycled bone after excision of tibia sarcoma in two cases. Plate and screws were used for osteosynthesis of the tibia or talus, vascularized fibula, and pasteurized bone. Microsurgery is not necessary for this reconstruction technique. Two patients who underwent this technique have obtained good functional results without tumor relapse 5 and 6 years after operation. The technique produced excellent results with regard to tibial reconstruction in these cases. We found it to be simple, speedy, safe, and a low cost technique by use of recycled bone.


*Sarcoma*, June/September 2004, *VOL. 8, NO. 2/3, 97–102*

Taylor & Francis healthsciences

**TECHNICAL NOTE**

### Reconstruction with ipsilateral fibula transfer with pasteurized bone after excision of bone sarcoma of the tibia

TOSHIFUMI OZAKI[1], KAZUO FUJIWARA[1], TOSHIYUKI KUNISADA[1], TATSUO ITO[1], AKIRA KAWAI[2], HAJIME INOUE[1]

[1]*Department of Orthopaedic Surgery, Okayama University Hospital, 2-5-1 Shikata-cho, Okayama 700-8558, Japan*
[2]*Department of Orthopaedic Surgery, National Cancer Center Hospital, Tsukiji, Chuo-ku, Tokyo 104-0045, Japan*

**Abstract**
We report a technique of implantation of the ipsilateral vascularized fibula with pasteurized recycled bone after excision of tibia sarcoma in two cases. Plate and screws were used for osteosynthesis of the tibia or talus, vascularized fibula, and pasteurized bone. Microsurgery is not necessary for this reconstruction technique. Two patients who underwent this technique have obtained good functional results without tumor relapse 5 and 6 years after operation. The technique produced excellent results with regard to tibial reconstruction in these cases. We found it to be simple, speedy, safe, and a low cost technique by use of recycled bone.

### Introduction

Tumor area in bone and its extraskeletal involvement can currently be clearly recognized with a careful preoperative radiological evaluation, including magnetic resonance (MR) imaging.[1] Improved imaging techniques[2] and preoperative chemotherapy[3,24] permit limb-sparing surgeries to be carried out safely in patients with bone and soft tissue tumors. Good long-term functional results have been reported after reconstruction of large bone defects using vascularized fibular grafts after tumor resection.[5] The bone union rate in one report was 81% (130 of 160 patients) at an average of 42 months after vascularized bone transfer.[6] However, lower extremity reconstructed with only a vascularized fibula apparently is too weak to withstand the mechanical stresses of full weight bearing before union or thickening of the grafted fibula. When a pasteurized bone shell is implanted around a vascularized fibula, the recycled bone can strengthen the fibula and bone junction, and protect the fibula from mechanical failure. Moreover, if ipsilateral vascularized fibula from the affected site is available, the operative invasion can be limited to only one leg. We report a technique of such a reconstruction of the tibial defects after tumor excision of the tibia in two cases. The ipsilateral vascularized fibula was transferred centro-medially, and pasteurized tibial bone was implanted as an autograft shell with plate and screws. The procedure is a modification of a technique originally described by Capanna *et al.*[7] and Wuisman *et al.*[8]

### Operative technique

The tibial tumor is excised with a wide margin. The attached soft tissue of the resected bone including the tumor is removed. Tumor resection and graft preparation procedures must be done on tables other than the instrumentation or operation table to prevent tumor contamination. A massive pasteurized bone graft is prepared from the resected tibial portion by treatment in physiological saline with povidone iodine (concentration, 3000 mg/1000 mL) at 60°C for 30 min.[10] The pasteurized bone then is reamed. One-third of the circumference of the pasteurized bone is excised longitudinally to allow implantation of the vascularized fibula in the cavity of the shell. Two-thirds of the tibia is retained for external support of the vascularized fibula. The ipsilateral fibula, which is usually cut 4–5 cm longer than the resection, is harvested with its periosteal cuff and peroneal vessels. The ends of

Correspondence to: Toshifumi Ozaki, Department of Orthopaedic Surgery, Okayama University Hospital, 2-5-1 Shikata-cho, Okayama 700-8558, Japan. Tel.: +81-86-235-7273; Fax: +81-86-223-9727; E-mail: tozaki@md.okayama-u.ac.jp

1357-714X print/1369–1643 online © 2004 Taylor & Francis Ltd
DOI: 10.1080/13577140400017794

the vascularized fibula are inserted a few centimeters into the medullary canal of the residual tibia, and 5 mm into the talus. The meta-diaphyseal intercalary pasteurized bone is implanted around the fibula with the vascular pedicle emerging from the split side of the pasteurized bone. The fibular blood flow is confirmed by the bleeding at the end of the bone before osteosynthesis.

### Case report

*Case* 1

A 45-year-old man had a sudden onset of left lower leg pain while he was playing baseball. A radiograph of the left lower leg revealed a pathological fracture of the tibia and he was referred to the authors' hospital. The patient's medical history and family history were unremarkable. The patient presented with swelling and tenderness of the left lower leg. Plain radiographs revealed an osteolytic shadow and a fracture at the distal metaphysis of the left tibia (Fig. 1). Bone scan showed an abnormal uptake from the shaft to the distal tibia. A digital subtraction angiograph showed increased vascularity of the vessels in the lesion. MR images showed a low-signal intensity tumor with an extraskeletal tumor projection on T1-weighted images (Fig. 2a) and a high-signal intensity on T2-weighted images. Gadolinium-diethylenetriaminepentaacetic acid (DTPA)-enhanced T1-weighted images showed imhomogeneous enhancement of the tumor (Fig. 2b). Open biopsy was performed; the diagnosis was leiomyosarcoma. Two cycles of preoperative chemotherapy ifosphamide, doxorubicin, and dacarubazine (MAID)[9] were administered. Preoperative intra-arterial administration of cisplatin (150 mg) was done twice. Preoperative MR images made after chemotherapy showed tumor limited to the tibia.

The tumor was excised with a wide margin. The resected bone was removed for pasteurization. The fibula was cut 5 cm longer than the resected tibia. After dividing the intraosseous membrane, the peroneal vessels were identified. The fibula was not released, but with the muscles, it was moved anteromedially. The proximal end was inserted in the residual tibia and the distal end was inserted in the talus. The pasteurized bone was implanted around the fibula. A long plate with seven screws was used for osteosynthesis.

The bone union was rapid, and partial weight bearing began 12 weeks after operation. At 6 months, full weight bearing was permitted. Twelve months after operation, the junction between the recycled bone and host bone had almost healed. The patient, a postman, had resumed his work, which included riding a motor bike. At 5 years after the operation, the junction was completely healed (Fig. 3). The leg length discrepancy was 3 cm. Evaluation scores at last follow-up, according to the method of Enneking *et al.*,[11] were 5 in pain, 4 in function, 4

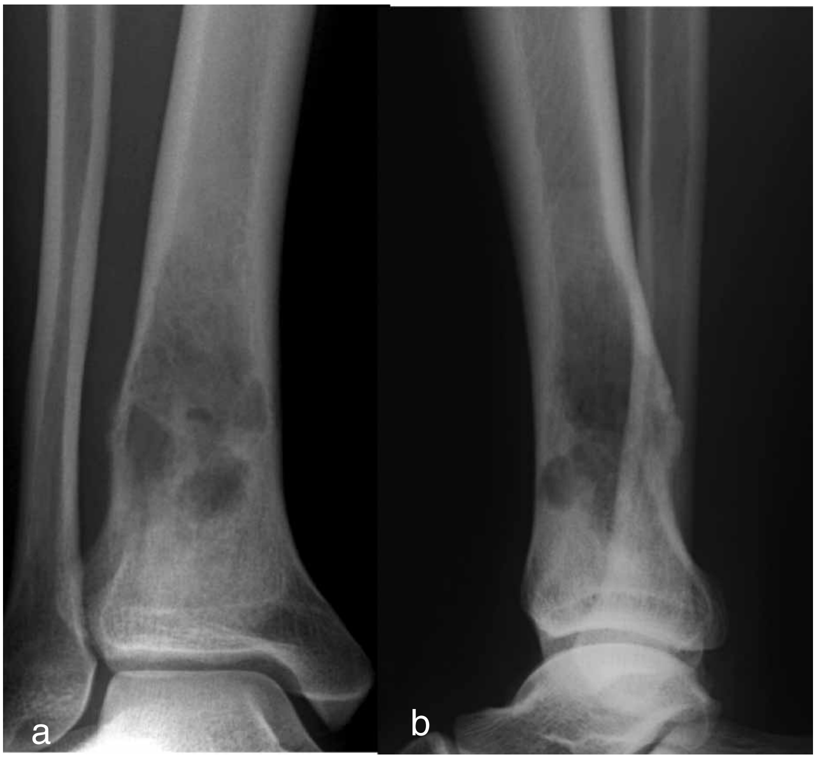

Ozaki T, et al.

**Fig. 1.** *Case* 1. *Plain radiograph shows a radiolucent area with pathological fracture in the distal tibia. (a) Antero-posterior view; (b) lateral view.*

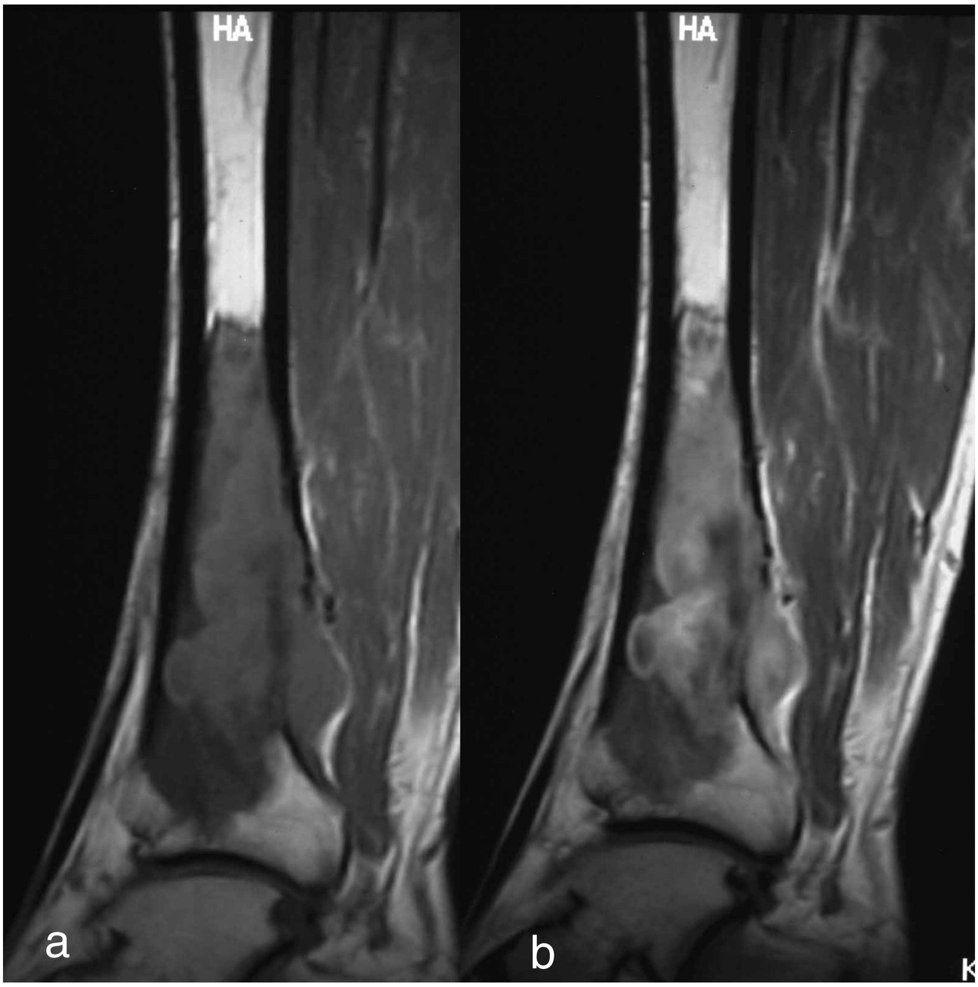

**Fig. 2.** *Case* 1. *(a) T1-weighted magnetic resonance (MR) imaging showed a low-signal intensity tumor with extra-skeletal projection. (b) Gadolinium-diethylenetriaminepentaacetic acid (DTPA)-enhanced T1-weighted images showed an inhomogeneously enhanced tumor.*

emotional acceptance, 5 support, 5 for walking, 4 for gait; 27 (90%) in total.

*Case* 2

A 12-year-old boy had right lower leg pain without trauma. His medical history indicated that he had had red cell aplasia 1 month after birth and had received steroid therapy. His family history indicated that his mother had colon cancer. The patient's lower leg pain increased gradually, and night pain developed. Abnormal findings were identified on plain radiographs, and he was referred to the authors' institute where he presented with tenderness on the proximal part of the right lower leg.

Plain radiographs showed a periosteal reaction of the posterior diaphysis and a sclerotic shadow in the meta-diaphysis of the right tibia (Fig. 4a). MR images showed a low signal intensity area of 15 cm on T1-weighted images (Fig. 4b) and a high signal intensity area on T2-weighted images in the diaphysis of the right tibia. A bone scan showed an abnormal high-uptake in the right meta-diaphysis of the tibia. Histological diagnosis indicated a high suspicion of benign tumor with a small possibility of osteosarcoma. There was no extra-skeletal tumor protrusion, and the diagnosis was unclear.

Tumor excision with a wide surgical margin without preoperative chemotherapy was performed in this patient. The residual epiphyseal bone segment measured 2 cm in thickness. After tumor excision, the vascularized fibula, which was 4 cm longer than the tibia defect, was transferred antero-medially. The vascularized fibula was inserted in the pasteurized resected tibia (Fig 4c), and both ends of the fibula were inserted in the residual tibia. Osteosynthesis was by means of metal plates with screws. Histological diagnosis after examination of the resected specimen was a low-grade osteosarcoma. Postoperative chemotherapy was administered according to the modified NECO95J protocol.[4] Partial weight bearing was started 11 weeks after operation. Full weight bearing was permitted from 3 months after operation. To date, the patient has survived more than 5 years without relapse after the operation. He can walk without external support (Fig. 5a). The bone union is excellent (Fig. 5b). The leg length discrepancy was 3 cm. Evaluation

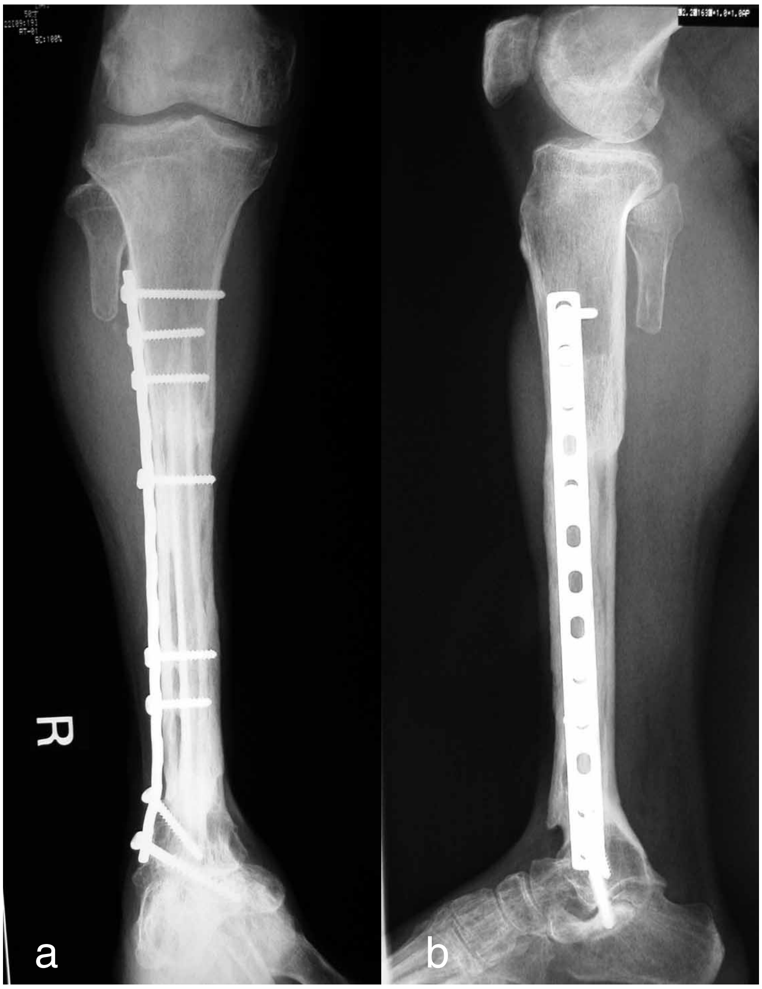

Ozaki T, et al.

**Fig. 3.** *Case* 1. *Radiograph taken at 5 years after operation. The junction site of bone healed completely. (a) Antero-posterior view; (b) lateral view.*

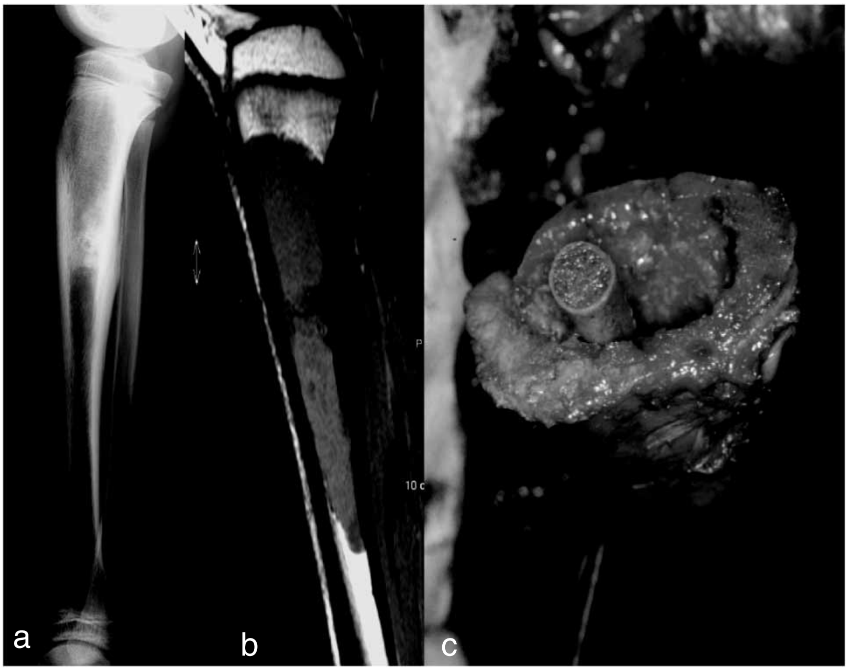

Ozaki T, et al.

**Fig. 4.** *Case* 2. *(a) Radiograph showed periosteal reaction of the posterior proximal meta-diaphysis of the tibia and sclerotic shadow in the proximal meta-diaphysis of the right tibia. (b) Magnetic resonance images showed a low signal intensity area (T1-weighted images). (c) The fibula end protrudes from the pasteurized tibia.*

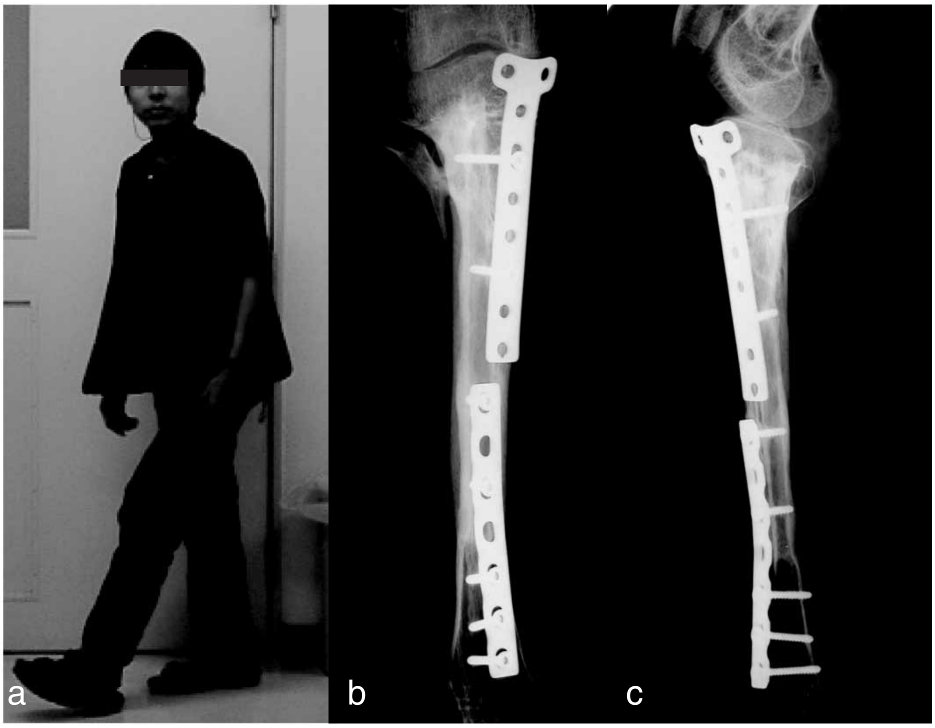

Ozaki T, et al.

**Fig. 5.** *Case 2. Five years after operation. (a) The patient, a high-school student at age 17 years, is shown using the knee brace. However, he can walk without further external supports. (b) Anterior-posterior view of plain radiograph. (c) Lateral view of plain radiograph.*

scores at last follow-up, according to the method of Enneking *et al.*,[11] were 5 for pain, 3 for function, 4 for emotional acceptance, 4 for support, 4 for walking, 4 for gait; 24 (80%) in total.

### Discussion

Cappana *et al.*, in 1991, reported that contralateral vascularized fibula anastomosis was performed after excision of the tibial sarcoma.[7] Their reconstruction procedure was characterized by a combination of an external allograft with an inner, vascularized free fibular flap.[7,12] In 1996, Wuisman *et al.* reported ipsilateral vascularized fibula transfer with implantation of allograft after excision of tibial sarcoma.[8] The author of the current report published the results of ipsilateral vascularized fibula and implantation of allograft shell in 12 cases.[13] The subsequent debate on the relative usefulness of the ipsilateral fibula graft versus the contralateral fibula graft continues.

Ipsilateral fibula transfer is an easy technique that can be completed in a relatively short operation time, and does not require vessel anastomosis. Volume reduction of the lower leg due to antero-medial shift of the fibula facilitates skin closure after tumor excision. However, careful control of alignment at the operative site is necessary to prevent malalignment of the lower leg. In cases of vascularized fibula transfer, valgus deformity of the donor site ankle is known to occur after fibulectomy,[14,15] especially in children younger than 10–12 years.[16,17] A tibiofibular syndesmotic screw efficiently prevents ankle valgus.[5]

We think that the fibula alone is inadequate to withstand compression and torsion stresses of weight bearing. And it is thought that a shell implant with plate and screws is a reasonable structural supplement for the fibula. Internal plate fixation is preferable to external fixation for osteosynthesis. In a previous report,[13] patients with fixation by screws or Kirschner wire had fracture or deformity of the junction, but patients with plate fixation had no such complications at the junction. A long plate with several screws is better than a minimal osteosynthesis using small fragment screws.[12] The risk of infection may be increased because patients usually receive postoperative chemotherapy and the pasteurized bone is not as strong as against infection. However, pasteurized bone, in general, has fewer complications and faster bone union than allograft bone does.[10,13]

Both cases in the current report illustrate reconstruction of the tibia with vascularized fibula transfer and pasteurized bone. The authors consider the method to be excellent for tibial reconstruction because it is a simple, speedy, and safe technique with an economical use of recycled bone.

### References

1. Spina V, Torricelli P, Montanari N, Manfrini M, Picci P, Sangiorgi L, *et al.* The epiphyseal involvement of metaphyseal bone sarcomas in patients with fertile growth plates. A magnetic resonance assessment. *Radiol Med* (*Torino*) 1994; 88(3): 190–7.

2. Ozaki T, Inoue H, Taguchi K, Sugihara S. Gadolinium-DTPA enhanced magnetic resonance imaging of bone and soft tissue sarcomas in comparison with pathological findings. *Acta Med Okayama* 1992; 46(6): 471–7.
3. Bielack S, Kempf-Bielack B, Schwenzer D, Birkfellner T, Delling G, Ewerbeck V, *et al.* [Neoadjuvant therapy for localized osteosarcoma of extremities. Results from the Cooperative osteosarcoma study group COSS of 925 patients.] *Klin Padiatr* 1999; 211(4): 260–70.
4. Isu K, Yamawaki S, Beppu Y, Umeda T, Kawaguchi T, Tatezaki S, *et al.* Prognostic results from multi-institute study of adjuvant chemotherapy for osteogenic sarcoma. *J Jpn Orthop Assoc* 1999; 73(Suppl): 1134–5.
5. Fragniere B, Wicart P, Mascard E, Dubousset J. Prevention of ankle valgus after vascularized fibular grafts in children. *Clin Orthop* 2003; 408: 245–51.
6. Han CS, Wood MB, Bishop AT, Cooney WP, 3rd. Vascularized bone transfer. *J Bone Joint Surg Am* 1992; 74(10): 1441–9.
7. Capanna R, Bufalini C, Campanacci M. A new technique for reconstructions of large metadiaphyseal bone defects. A combined graft (allograft shell plus vascularized fibula). *Orthop Traumatol* 1993; 2: 159–77.
8. Wusiman P, Gruenert J, Langer M. Ipsilateral, pedicled fibula transfer in the treatment of malignant tibia tumor. *Op Orthop Traumatol* 1996; 8(2): 142–52.
9. Kawai A, Naito N, Sugihara S, Ozaki T, Morimoto Y, Inoue H. Mesna, adriamycin, iifosphamide, dacarbazine (MAID) chemotherapy for high grade soft tissue sarcomas. *Orthop Surg* 2000; 51(5): 509–13.
10. Manabe J, Kawaguchi N, Matsumoto S. Pasteurized autogenous bone graft for reconstruction after resection of malignant bone and soft tissue tumors: imaging features. *Semin Musculoskelet Radiol* 2001; 5(2): 195–201.
11. Enneking WF, Dunham W, Gebhardt MC, Malawar M, Pritchard DJ. A system for the functional evaluation of reconstructive procedures after surgical treatment of tumors of the musculoskeletal system. *Clin Orthop* 1993; 286: 241–6.
12. Manfrini M, Gasbarrini A, Malaguti C, Ceruso M, Innocenti M, Bini S, *et al.* Intraepiphyseal resection of the proximal tibia and its impact on lower limb growth. *Clin Orthop* 1999; 358: 111–9.
13. Ozaki T, Hillmann A, Wuisman P, Winkelmann W. Reconstruction of tibia by ipsilateral vascularized fibula and allograft. 12 cases with malignant bone tumors. *Acta Orthop Scand* 1997; 68(3): 298–301.
14. Weiland AJ, Moore JR, Daniel RK. Vascularized bone autografts. Experience with 41 cases. *Clin Orthop* 1983; 174: 87–95.
15. Weiland AJ, Weiss AP, Moore JR, Tolo VT. Vascularized fibular grafts in the treatment of congenital pseudarthrosis of the tibia. *J Bone Joint Surg Am* 1990; 72(5): 654–62.
16. Omokawa S, Tamai S, Takakura Y, Yajima H, Kawanishi K. A long-term study of the donor-site ankle after vascularized fibula grafts in children. *Microsurgery* 1996; 17(3): 162–6.
17. Dean GS, Kime RC, Fitch RD, Gunneson E, Urbaniak JR. Treatment of osteonecrosis in the hip of pediatric patients by free vascularized fibular graft. *Clin Orthop* 2001; 386: 106–13.
